# Abundance and seasonality of phoronid larvae in coastal temperate waters: More abundant than previously thought?

**DOI:** 10.1007/s10452-022-09982-6

**Published:** 2022-08-29

**Authors:** Maria McGuinness, Hannah Brownlow, Rob McAllen, Luke Harman, Damien Haberlin, Thomas K. Doyle

**Affiliations:** 1 School of Biological, Earth and Environmental Sciences, University College Cork, Distillery Fields, North Mall, Cork, Ireland; 2 MaREI, The SFI Research Centre for Energy, Climate and Marine, Beaufort Building, Environmental Research Centre, University College Cork, Ringaskiddy, Cork Ireland

**Keywords:** Phoronida, Biodiversity, Marine protected area, Larval abundance, Plankton sampling

## Abstract

In zooplankton surveys, many smaller taxa or species considered less important are often overlooked. One such example is the actinotrocha larvae of phoronid worms that are rarely quantified in zooplankton samples yet may play important roles in marine food webs. To gain a better understanding of phoronid ecology in coastal waters, we retrospectively analysed 145 plankton samples collected from two coastal sites in Ireland (Lough Hyne and Bantry Bay). Samples were collected using plankton nets from depths of 20 and 40 m. Phoronids were present in 37.7% and 38.2% of samples, with mean abundances of 0.3 ± 0.5 ind. m^−3^ and 1.2 ± 2.8 ind. m^−3^, respectively, and were identified as *Phoronis muelleri* and *Phoronis hippocrepia*. Phoronids were present consistently each year from April to October at Lough Hyne and from February to October at Bantry Bay. Comparisons with other taxa in Lough Hyne show that abundances are similar to those of fish larvae (1.1 ± 1.8 ind. m^−3^) and echinoderm larvae (2.3 ± 4.4 ind. m^−3^). Examination of these samples from Irish waters suggests that phoronids are more abundant in temperate waters than previously reported.

**Supplementary Information:**

The online version contains supplementary material available at 10.1007/s10452-022-09982-6.

## Introduction

**Fig. 1:**
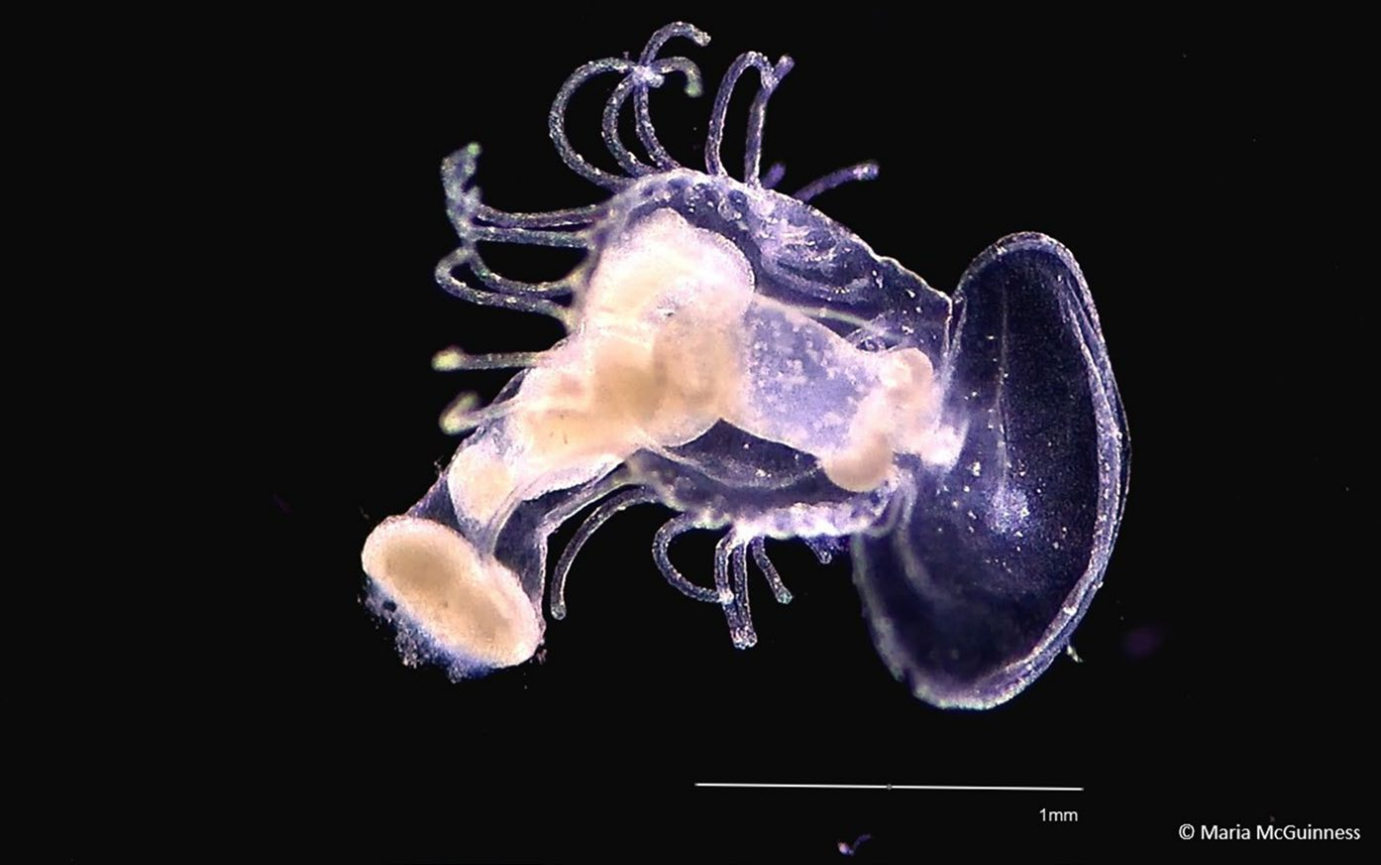
Image of *Phoronis muelleri* found at Lough Hyne

Accurate measures of biodiversity are an extremely important tool in conservation. However, in the marine ecosystem, it is estimated that only one third of species have been described (Appeltans et al. 2012). Even within the described species, many sampling methodologies are biased towards groups such as crustaceans which are highly abundant and commercially important, and thus, some non-crustacean species often escape being recorded in these surveys (Raskoff et al. 2005). This can lead to conclusions that such taxa are unimportant, rare and poorly described. An example of this is the phoronid worms (Phylum Phoronida). Phoronida is a small phylum of filter-feeding marine worms which live in a cylindrical tube they have secreted themselves (Emig 1977). They have a pelagic larval stage (excluding *Phoronis ovalis*), known as the actinotrocha, which can spend a few weeks to a few months living in the plankton before settling on a suitable substrate where it metamorphoses into a sessile adult (Fig. 1; Temereva and Neretina 2013; Emig 1982). Morphologically, the actinotrocha stage is very different to the adult stage and so was originally believed to be a separate taxonomic group entirely. This had led to a mismatch in taxonomic records, with three times as many actinotrocha described as adults (Temereva and Nemertina 2013). There are currently 13 accepted species as listed in the World Register of Marine Species in two genera: *Phoronis* and *Phoronopsis* (WoRMS 2022). Phoronids have a global distribution and are described as being euryhaline, eurythermal and resistant to the effects of red tides, suggesting they are highly adaptable (Emig 1982). Adult phoronids can occur in large aggregations, as high as 91,000 individuals per m^2^ (ind. m^−2^) (Ocharan 1978). Despite this and worldwide extensive plankton studies, actinotrocha abundances are rarely reported in the literature. Furthermore, they are often collected in plankton samples but not reported (Damien Haberlin, pers. comms). There are currently no estimates of actinotrocha abundances in Irish waters. To address this, we decided to retrospectively analyse 145 plankton samples from two coastal sites in Ireland to determine actinotrocha abundance and compare this with other well-known taxa.

## Materials and methods

### Sampling sites

**Fig. 2:**
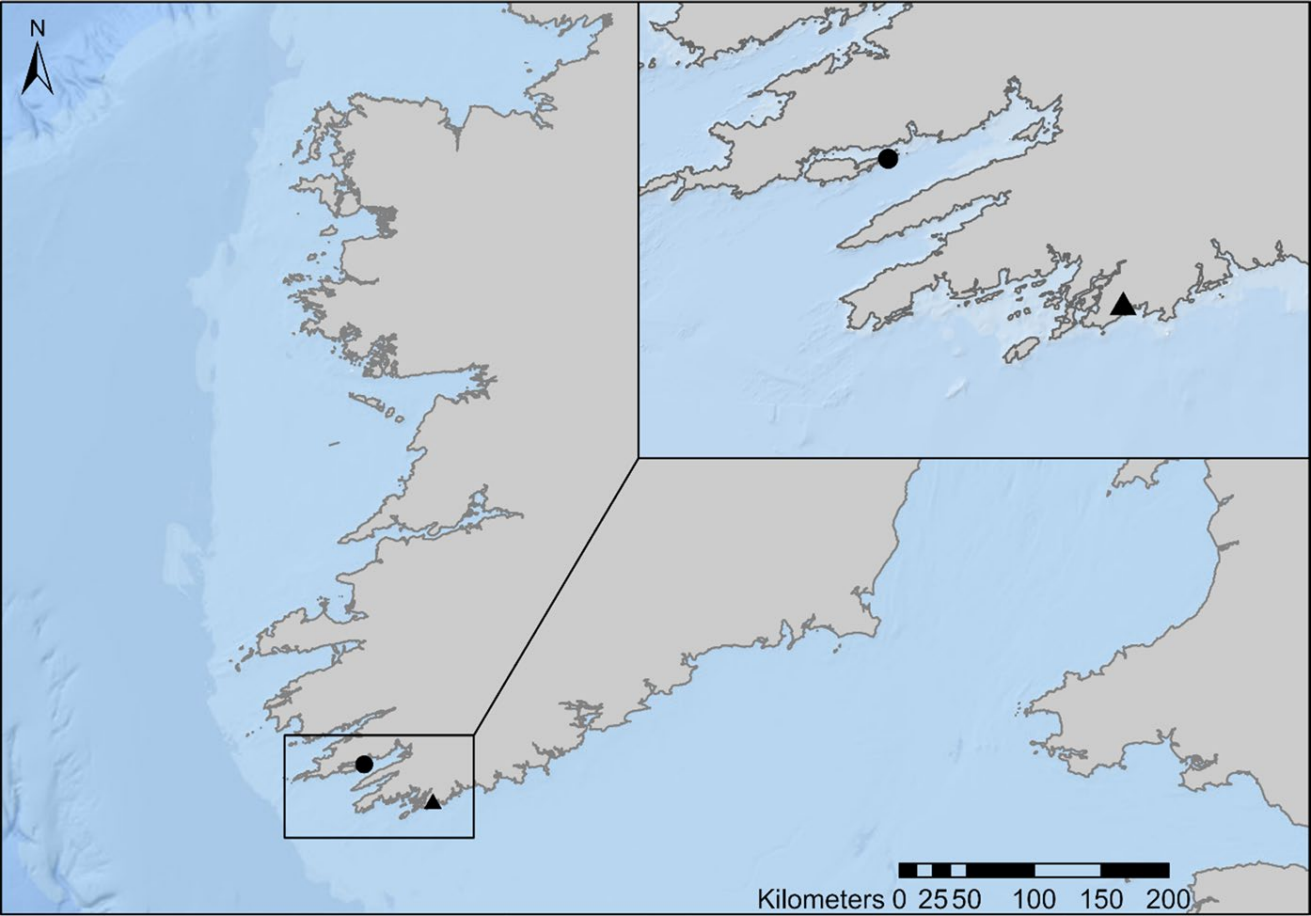
Map of study sites. The triangle marks Lough Hyne and the circle marks Bantry Bay. Scale bar shown at bottom right of the map

Plankton samples from Lough Hyne and Bantry Bay were analysed for the presence of actinotrocha (Fig. 2). Sampling sites were chosen based on existing plankton monitoring programs. Lough Hyne is a semi-enclosed marine lake located in southwest County Cork. It is 0.8 km long by 0.6 km wide and is Ireland’s only statutory marine reserve. Bantry Bay is a large natural bay located in southwest County Cork. It is 35 km long, 3 km wide at the head and 10 km wide at the entrance.

### Sample collection and analysis

In Lough Hyne, plankton samples were collected in the years 2018–2021. Three different plankton sample collections were analysed. On 31/05/2018, discrete depth plankton samples at 10 m intervals to a maximum depth of 40 m were collected. In 2019, vertically integrated plankton samples from 20 m depth were collected on three sampling occasions in May and June. Starting in June 2020, vertically integrated plankton samples from 40 m depth were collected biweekly in the summer months and monthly in the winter months until June 2021. All samples were taken at a fixed anchored buoy in the western trough of the Lough. A plankton net with an opening ring diameter of 0.5 m, length 2 m and mesh size 200 µm was used for sample collection. An opening/closing mechanism was used for discrete depth samples collected on 31/05/2018. A total of 77 samples were collected.

In Bantry Bay, vertically integrated plankton samples from 20 m depth were collected biweekly in the summer months and monthly in the winter months in the years 2020 and 2021. All samples were taken at a fixed anchored buoy located ~ 50 m from a salmon farm in Bantry Bay. A plankton net with an opening ring diameter of 0.5 m, length 1.5 m and mesh size 200 µm was used for sample collection. A total of 68 samples were collected.

All samples were immediately preserved in 4% formalin solution and stored prior to analysis. Samples were analysed using a Zeiss dark-field stereomicroscope. Density was calculated by dividing total count by volume of water sampled. From 2018 to end 2020, this was calculated by assuming a cylindrical column of water was sampled, based on the net diameter and towing distance. Flowmeters were used from the beginning of 2021. Multiple taxa were counted for Lough Hyne, while only actinotrocha were counted in Bantry Bay. Actinotrocha were identified with the help of Dr Elena N. Temereva. Data visualisation was conducted in R (version 4.0.5).

## Results

### Lough Hyne

**Fig. 3:**
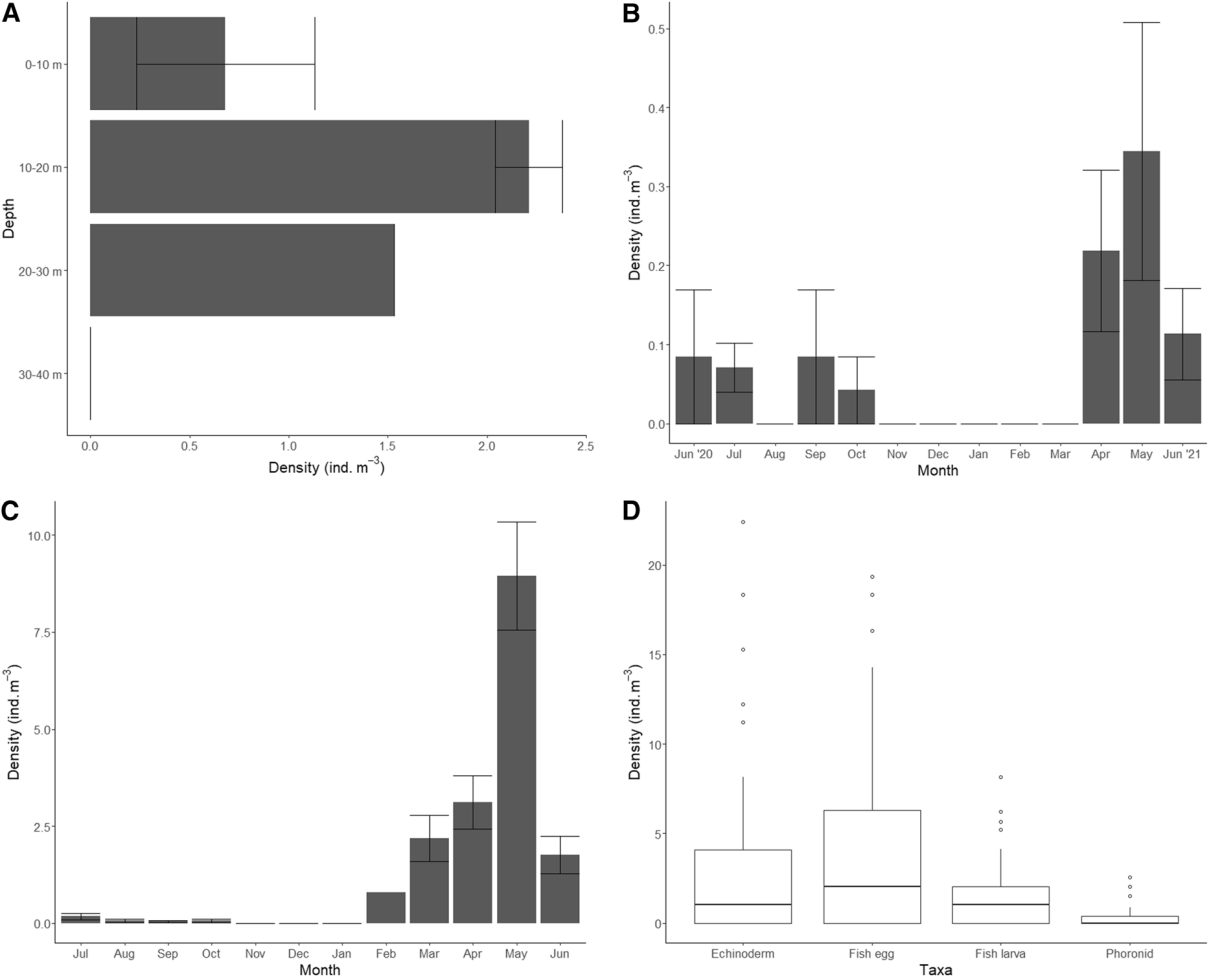
For plots **a**–**c**, bars show a mean value with error bars. **a** Phoronid larvae densities at discrete depths in Lough Hyne May 2018. **b** Phoronid larvae densities in Lough Hyne from June 2020 to June 2021. **c** Phoronid larvae densities in Bantry Bay from July 2020 to June 2021 **d** Densities of four taxa in Lough Hyne in the months April–October for all years sampled. Black lines represent the median. The box represents the interquartile range. Circles represent outliers

Of the 77 samples analysed, 29 samples (37.7%) contained at least one actinotrocha. These were identified as *Phoronis muelleri* (Fig. 1) and *Phoronis hippocrepia* (Supplementary information). A total of 65 actinotrocha were counted. Actinotrocha were present in all years sampled (2018–2021). The 2018 discrete depth samples revealed that abundances were similar at all depths except for the 30–40 m strata where no actinotrocha were present (Fig. 3a). In 2020, they were present from June until October and absent during winter months. In 2021, they were present in April, May and June (Fig. 3b, Table 1). The mean density (ind. m^−3^) of actinotrocha across all samples was 0.3 ± 0.5 (mean ± s.d.) with a maximum of 2.6 ind. m^−3^ in the 10–20 m strata on 31/05/2018 (range = 0–2.6; median = 0). The abundance range of other taxa was as follows: the mean density of fish eggs was 3.7 ± 5.4 ind. m^−3^ (range = 0–19.4; median = 1.0; frequency = 55.1%; seasonality = February–December), fish larvae 1.1 ± 1.8 ind. m^−3^ (range = 0 – 8.2; median = 0.2; frequency = 53.6%; seasonality = February–December), hydromedusae 423.4 ± 546.3 ind. m^−3^ (range = 2.7–2697.3; median = 177.2; frequency = 100%; seasonality = year-round) and echinoderm larvae 2.3 ± 4.4 ind. m^−3^ (range = 0–22.4; median = 0.38; frequency = 53.6%; seasonality = March–December; Fig. 3d based on season when actinotrocha are present). The most abundant component of the plankton, copepods, had a mean density of 10,686.3 ± 11,912.6 ind. m^−3^ (range = 68.4–53,024.7; median = 7094.9; frequency = 100%; seasonality = year-round).

**Table 1:** Monthly mean temperatures (°C) at 5 m depth in Bantry Bay and Lough Hyne from July 2020 to June 2021

	Lough hyne	Bantry bay
JuL 2020	14.8	15.0
Aug	14.9	17.1
Sep	14.9	14.7
Oct	12.9	11.5
Nov	11.9	10.8
Dec	10.6	10.0
Jan 2021	9.3	8.6
Feb	8.3	8.5
Mar	10.5	8.7
Apr	10.7	10.0
May	12.7	11.2
Jun	14.0	13.8

### Bantry Bay

Of the 68 samples analysed, 26 samples (38.2%) contained at least one actinotrocha. These were identified as *Phoronis muelleri*. A total of 205 actinotrocha were counted in these samples. The mean density of actinotrocha in the samples was 1.2 ± 2.8 ind. m^−3^ with a maximum of 14.2 ind. m^−3^ in May 2021 (range = 0 – 14.2; median = 0). Actinotrocha were present from July to October in 2020 and from February to June in 2021 (Fig. 3c, Table 1).

## Discussion

Phoronid worms have a global distribution, are highly adaptable, are an important component of some subtidal habitats and can reach very high densities on a variety of substrates (Emig 1982; Temereva et al. 2016; Temereva & Neklyudov 2018). While there is a substantial body of research on phoronids, there has been less of a focus on ecology and there is no current abundance estimate for their larvae, actinotrocha, in Irish coastal waters. Here, we have retrospectively analysed plankton samples from two coastal locations in Ireland and found that actinotrocha are relatively common, occurring in 37.7% and 38.2% of samples, respectively. Furthermore, these have been identified as consisting of two species: *P. muelleri* and *P. hippocrepia*.

Within the plankton community, it was notable that the abundance of actinotrocha in Lough Hyne (0.3 ± 0.5 ind. m^−3^) is comparable with fish larvae (1.1 ± 1.8 ind. m^−3^) and echinoderm larvae (2.3 ± 4.4 ind. m^−3^; Fig. 2c). However, the number of studies investigating all three of these taxa is extremely unbalanced. This raises the question of taxonomic chauvinism, a term coined by Bonnet et al. (2002), describing the overrepresentation of certain taxa in the literature. Why is it that there are far more studies on fish larvae and echinoderm larvae? Likely because they are considered commercially important, “charismatic” and better known to us. In contrast, phoronids are less prominent in the literature despite being a potentially influential component of coastal ecosystems. Firstly, adult phoronids are filter feeders, with the potential to impact water quality by filtering out particles with a size range of 1.2–12 µm (Temereva and Malakhov 2010). This includes phytoplankton, detritus and larger bacteria. Secondly, as encrusting organisms they bore into a substrate where they remain attached for the remainder of their life cycle, so can be considered habitat modifying organisms and can affect the distribution of other species in an area. Indeed, *Phoronopsis harmeri* was seen to positively impact the abundance of several infaunal species increasing the overall species richness in Bodega Harbour, California (Larson et al. 2009). Finally, phoronids that burrow into soft structures such as *Zostera* beds have been seen to reduce erosion of the sediment by stabilising it and its infaunal community (Emig et al. 1977; Emig 1982). It is clear phoronids are beneficial in many marine systems, potentially including Bantry Bay and Lough Hyne.

The abundances of actinotrocha reported here (Lough Hyne mean: 0.3 ± 0.5 ind. m^−3^; Bantry Bay mean: 1.2 ± 2.8 ind. m^−3^) are comparable to those found in the Caribbean (0.2 ± 0.3 ind. m^−3^) where *P. hippocrepia* and *P. muelleri* are also found, along with six other phoronid species (Collin et al. 2019). However, the abundances are much lower than those reported in the Indo-Pacific, which has been suggested as a hotspot for phoronid biodiversity (Temereva et al. 2016). 3490 ind. m^3^ of actinotrocha were recorded in the Sea of Japan in November 2005, but unfortunately, the species was not recorded (Omelyanenko and Kulikova 2011). Although less abundant, the seasonality of actinotrocha in our study was longer than in the above study. Actinotrocha were present from February to October in Bantry Bay and from April to October in Lough Hyne with peak abundances in May for both sites. In the Sea of Japan, actinotrocha were present from July–December (Omelyanenko and Kulikova 2011). This seasonality is also slightly different to that documented in other North-East Atlantic areas such as Sweden where actinotrocha were present from August to December (Moksnes et al. 2014). This implies that phoronids may have a longer spawning period in Irish waters.

Previous reports of phoronids in Ireland are scarce and have mostly only recorded presence rather than abundance or seasonality. In Lough Hyne, the presence of *P. hippocrepia* has been recorded by Renouf (1939) and Kitching (1987). Santagata and Cohen (2009) reported the presence of *P. ovalis* in the Irish Sea based on DNA evidence. Byrne (1995) recorded single specimens of unknown actinotrocha in Galway Bay at two time points. Rodhouse and Roden (1987) recorded the biomass of adult *P. muelleri* in Killary Harbour (3.5–497.2 mg m^−2^ AFDW), while Silke et al (2005) recorded the biomass of adult *P. hippocrepia* in Killary Harbour (0.06–0.50 ind. 0.01 m^−2^). There are no data for either adults or actinotrocha in Bantry Bay. Otherwise, their presence in the plankton has been largely ignored. This study showed the consistent presence of phoronids at two different coastal locations, and we suggest they may be present in many more locations around Ireland but have not been recorded. They are certainly not the only ‘rare’ taxa which have been historically under-recorded in plankton research. Increased understanding of the breadth of marine biodiversity is essential, especially as protecting marine ecosystems, and biodiversity becomes ever more urgent. It is vital that future studies report on not just the seemingly important species, but also the seemingly rare ones too.

## Supplementary Information

Below is the link to the electronic supplementary material.

Supplementary file1 (XLSX 24 KB)

Supplementary file2 (PNG 146 KB)

## Data Availability

All data generated or analysed during this study are included in this published article [and its supplementary information files].
