# Supplementary figures and images for: Abundance and seasonality of phoronid larvae in coastal temperate waters: More abundant than previously thought?
Source: Aquat Ecol. 2022 Aug 29;56(4):1315–21. doi: 10.1007/s10452-022-09982-6 (PMC9618493; doi:10.1007/s10452-022-09982-6)

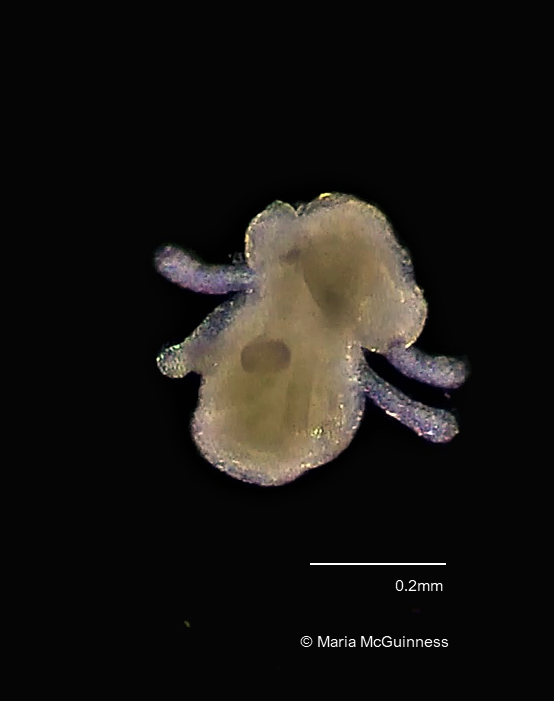

Supplement: Supplementary file 2 — Supplementary file2 (PNG 146 KB) [file 10452_2022_9982_MOESM2_ESM.png]
